# Interface Engineering Enables High-Performance Sb Anode for Sodium Storage

**DOI:** 10.3390/nano13020254

**Published:** 2023-01-06

**Authors:** Chang Liu, Xin Fu, Shuzhen Liao, Guoqiang Zou, Hai Yang

**Affiliations:** 1 School of Chemistry and Chemical Engineering, Hunan Institute of Engineering, Xiangtan 411104, China; 2 College of Chemistry and Chemical Engineering, Central South University, Changsha 410083, China

**Keywords:** sodium-ion batteries, Sb, heterointerface engineering

## Abstract

Heterointerface engineering has been verified to be an effective approach to enhance the energy density of alkali-ion batteries by resolving inherent shortcomings of single materials. However, the rational construction of heterogeneous composite with abundant heterogeneous interfaces for sodium-ion batteries (SIBs) is still a significant challenge. Herein, inspired by density functional theory calculations, interface engineering can greatly decrease the energy bandgap and migration barrier of Na ions in Sb and Na_3_Sb phases, as well as enhance the mechanical properties. A porous heterointerface MOFC–Sb is fabricated by utilizing MOF-C as a support and buffer, exhibiting excellent electrochemical performances for sodium storage. The MOF-C–Sb anode with its rich heterointerface presents an improved electrochemical performance of 540.5 mAh g^−1^ after 100 cycles at 0.1 A g^−1^, and 515.9 mAh g^−1^ at 1.6 A g^−1^ in term of sodium storage, efficiently resolving the serious volume expansion issues of metal Sb. These results indicate the structural superiority of heterointerface-engineered structure and afford valuable information for the rational design and construction of Sb-based anode materials for high-performance electrochemical energy storage.

## 1. Introduction

Lithium-ion batteries (LIBs) can barely meet the cumulative requirements in low-speed electric vehicles and large-scale energy storage fields (e.g., grid storage) in the near future, in view of the extremely uneven distribution of lithium resources in the earth’s crust and the ever-rising price of lithium salts. Sodium-ion batteries (SIBs), delivering the same cell structure as LIBs, have been regarded as one of the most hopeful alternatives to LIBs by virtue of the rich sodium resources and similar electrochemical energy storage mechanism [1,2,3,4,5]. However, high-performance sodium-ion storage electrodes still present many challenges since the radius of Na^+^ is 35% larger than that of Li^+^, largely restricting the development of SIBs. The successful commercial graphite anode used in LIBs is difficult to insert Na^+^ into. So, improvement of high-performance anodes for sodium ions is urgently required for industrial applications of SIBs [6]. Driven by the good conductivity, high theoretical specific capacity, and relatively low energy storage potential (vs. Li/Li^+^) of metal anodes in LIBs, extensive efforts have been made to develop metal anodes (e.g., Sn, Sb, and Bi) for sodium storage, in which Sb is deemed to the most hopeful anode of SIBs by the reason of its high theoretical capacity of 660 mAh g^−1^ [7]. Nevertheless, the key problem obstructing the practical application of Sb anodes is due to its poor cyclability, which can be ascribed to the dramatic volume expansion during repeated sodiation, further bringing about the pulverization of the electrode and loss of electrical contact between Sb and the current collector [8,9,10,11]. The dimensional structure of matter has a great influence on its related properties [12,13,14,15]; hence, it is very important to develop feasible methods to alleviate the volume expansion during charging and discharging processes, and improve the mechanical properties of Sb for the practical application of Sb anodes.

In the past decade, great efforts have been made to resolve the pulverization and capacity decline of Sb electrodes. Momentous progress has been attained. Current strategies mostly focus on designing various nanostructures of Sb, such as Sb nanoparticles [16], porous Sb [17], leaf-like Sb [18], Sb nanospheres [19], and Sb nanosheets [20,21], which could modify reaction kinetics and enhance cycling stability. Among these strategies, nanostructured Sb anodes have been verified to be one of the most effective routes for relieving volumetric expansion of Sb anodes during cycling. Jiang et al. reported a chemical dealloying approach to the tunable preparation of multidimensional Sb nanostructures; the as-obtained Sb electrodes display a superior Na^+^ storage capacity of 620 mAh g^−1^ at 100 mA g^−1^, long-term cycle stability with 90.2% capacity retention after 100 cycles, and an excellent rate performance of 212 mAh g^−1^ at 6.4 A g^−1^ [8]. Although some progress has been made in the modification of antimony-based materials, their cycle life and electrochemical performance still need to be further improved. Heterointerface engineering in antimony-based materials has recently captured interest due to their abundant active sites and reconstructed electronic structure. Extensive efforts have demonstrated that heterointerface engineering can largely reduce the embedding and migration barriers of sodium ions, providing more active sites for sodium and, thus, hastening the charge transfer kinetics, which significantly augment the reversible capacity and rate capability.

Herein, inspired by density functional theory calculations that interfacial Sb–C engineering can not only largely decrease the energy bandgap from 2.2 eV to 0.89 eV and the reaction energy barrier of Sb and Na_3_Sb phases, but can also enhance the mechanical properties, porous heterointerface MOF-C–Sb is successfully obtained by utilizing MOF-C as a support and buffer, delivering excellent electrochemical performances for sodium storage. The MOF-C–Sb anode with its rich heterointerface shows an improved electrochemical performance of 540.5 mAh g^−1^ after 100 cycles at 0.1 A g^−1^, and 515.9 mAh g^−1^ at 1.6 A g^−1^ for sodium storage, efficiently resolving the serious volume expansion issues of metal Sb, demonstrating the structural superiority of the heterointerface-engineered structure and providing valuable information for the rational design and construction of Sb-based anode materials for high-performance electrochemical energy storage. In order to better understand this paper, the first part of this paper mainly introduces the research status of antimony-based materials, the second part mainly discusses the experimentation, the third part contains results and discussion, and the fourth part is the conclusion.

## 2. Experimental Section

### 2.1. Material Preparation

All chemical regents are of analytical grade and can be utilized without any further purification. Firstly, the modified method for preparing MOF-5 was adopted [22]. Briefly, 3.4 g Zn(NO_3_)_2_•6H_2_O and 0.6 g H_2_BDC (terephthalic acid) were dissolved in 45 mL anhydrous DMF under violent stirring. The solution was transferred to a Teflon-lined autoclave after complete dissolution. Next, the autoclave was sealed and heated at 120 °C for 24 h. After being cooled naturally at room temperature, washed with CH_2_Cl_2_ three times, and finally dried, the MOF-C material was obtained by a subsequent pyrolysis of MOF-5 at 1100 °C for 2h under an Ar atmosphere with a heating rate of 5 °C per minute. A total of 0.05 g as-obtained MOF-C was added to 40 mL glycol with a 30 min ultrasonic process. Then, 0.4 g SbCl_3_ was added to the solution. Subsequently, 40 mL mixed solution of NaOH (1.2 g) and NaBH_4_ (0.2 g) was dropped into the above solution under magnetic stirring at 60 °C for 2 h. The MOF-C–Sb composite was obtained by centrifugation, washed thoroughly with ethanol/water, and dried under vacuum. 

### 2.2. Materials Characterization

XRD (Rigaku D/max 2550, Rigaku, Tokyo, Japan), BET analysis (BELSORP-MINI II, Ankersmid, Samuel Morsestraat, The Netherlands), XPS characterization (ESCALab250, Thermo Fisher Scientific, Waltham, MA, USA), TGA (NETZSCH STA449F3, NETZSCH, Wittelsbacherstr, Germany) (the heating rate was 5 °C/min in an air atmosphere), Raman spectroscopy (Jobin–Yvon Lab RAM HR-800, HORIBA Jobin Yvon, Paris, France), and FTIR (FTIR (AVTA-TAR, 370, Nicolet, Green Bay, WI, USA) characterization were employed to study the structure and composition of the samples. SEM (FEI Quanta 200, Thermo Fisher Scientific, USA) and TEM (JEM-2100F instrument, JEOL, Tokyo, Japan) were conducted to detect the morphology of the as-prepared materials. 

### 2.3. Half-Cell Preparation and Electrochemical Measurement vs. Na 

The electrodes were made up of 70 wt% active material, 15 wt% conductive carbon (Super P), and 15 wt% binder carboxymethyl cellulose. Then, the mixture was dissolved in a defined amount of deionized water to generate slurry. After being stirred for 12 h, the electrode film was obtained after the mixture was evenly painted onto Cu film and dried overnight in a vacuum drying oven at 100 °C. The mass loading of active material was about 1.0-1.3 mg cm^−2^. Under an anhydrous and anaerobic atmosphere, CR2016-type coin half-cells were assembled inside a Braun glovebox with metallic sodium to be used as a counter electrode and polypropylene film was utilized as a separator (Celgard 2400), respectively. The 1 M NaClO_4_ solution with propylene carbonate as solvent was used as the electrolyte for SIBs. In galvanostatic charge/discharge tests, the electrochemical impedance performance and cyclic voltammetry (CV) measurements were collected through an Arbin battery cycler (BT2000) and a Solartron analytical instrument between 2.0–0.01 V.

### 2.4. DFT Calculations

Density functional theory (DFT) calculations were conducted by the Cambridge Serial Total Energy Package (CASTEP) module [23], in which the electron exchange-correlation potential was obtained by the Perdew–Burke–Ernzerhof (PBE) functional with generalized gradient approximation (GGA) [24], and the ultra-soft pseudo-potentials were employed. The kinetic energy cutoff was adjusted to 450 eV for the plane-wave basis set. Brillouin zone integration was sampled with 3 × 3 × 3, 4 × 4 × 2, and 2 × 2 × 1 Monkhorst–Pack mesh k-points for Sb, Na_3_Sb, and the interface, respectively. To evaluate the diffusion paths of Na^+^ in as-obtained materials, synchronous transit methods with completed linear synchronous transit (LST) and quadratic synchronous transit (QST) were utilized [25]. The convergence tolerances were set to be 2 × 10^−5^ eV per atom for energy, 2 × 10^−3^ Å for maximum displacement, and 0.05 eV/Å for maximum force. All of the structures were fully optimized and relaxed to the ground state.

## 3. Results and Discussion

**Figure 1:**
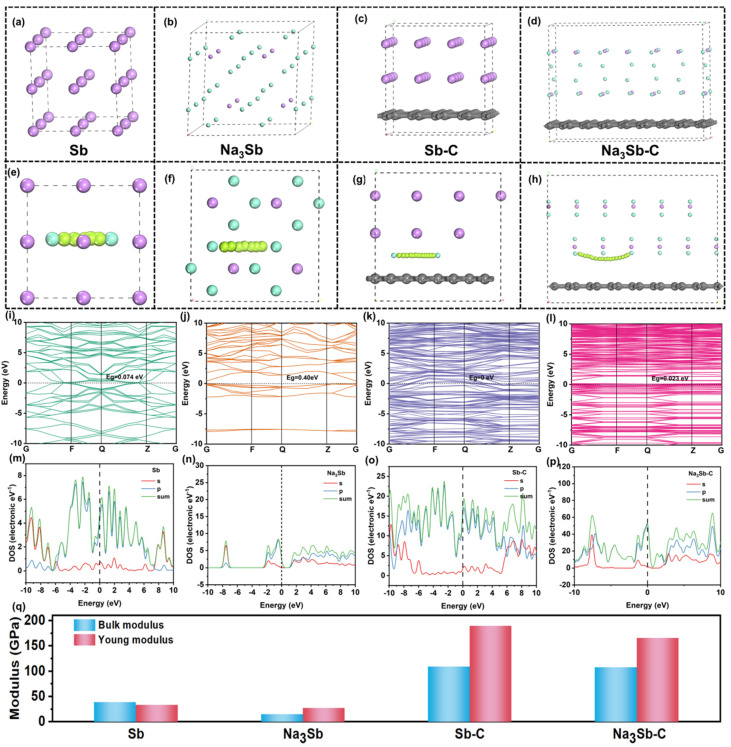
The computational models for (**a**) Sb, (**b**) Na_3_Sb, (**c**) Sb−C, (**d**) Na_3_Sb−C, and Na ion migration path in Sb (**e**), Na_3_Sb (**f**), Sb−C (**g**), Na_3_Sb−C (**h**), respectively (the pink, black, and lime green spheres are Sb, C, and Na atoms, green spheres are the migration path of Na). The calculated bandgap structures and partial density of states for (**i**,**m**) Sb, (**j**,**n**) Na_3_Sb, (**k**,**o**) Sb−C, and (**l**,**p**) Na_3_Sb−C. (**q**) The Bulk modulus and Young’s modulus of related materials.

DFT calculations are first performed and the model structures of the theoretical calculations are shown in Figure 1a–d. The atoms of Sb and Na_3_Sb are arranged in a regular orderly pattern and the coating of carbon has no change in its alignment. Furthermore, the migration paths of sodium ions have been presented in Figure 1e,h. The migration barrier of Na ions in the Sb–C interface is 0.93 eV, which is obviously lower than that in Sb (2.22 eV), indicating that the transfer rate of Na ions in Sb–C materials is higher than that in Sb crystals. After the insertion of Na, the Sb transforms into Na_3_Sb, so the migration barriers of Na ions in Na_3_Sb and Na_3_Sb–C are assessed, in which the migration barrier of Na ions in the Na_3_Sb–C interface is 0.89 eV, which is also lower than that in Na_3_Sb–C (1.24 eV), demonstrating that the introduction of the interface can efficiently reduce the ion migration barrier, so as to effectively improve the ion migration rate in electrode materials. The energy bandgap of Sb–C (0 eV, Figure 1i) is lower than that of pure Sb (0.074 eV, Figure 1k), suggesting a better electronic effect from the interfacial carbon. As shown in Figure 1j,l, the insertion of Na^+^ into both Sb and Sb–C induces an increase in the band gap, indicating that the electronic conductivity reduces as the amount of inserted Na^+^ increases. Consequently, the energy bandgap of Na_3_Sb–C is as small as 0.023 eV, which is far smaller than that of Na_3_Sb (0.40 eV) and Sb, delivering metal-like properties with good electrical conductivity, showing that the interface can efficiently enhance the electrical conductivity. The density of states (DOS) of Sb shows no significant difference compared to that of Sb–C, since their main phase is metallic and both valence band maximum (VBM) and conduction band minimum (CBM) are close to 0 (Figure 1m,o). However, the difference between VBM and CBM is lower in Na_3_Sb–C than in Na_3_Sb, further confirming the fast reaction kinetics of Na^+^ in Sb–C, which is consistent with the energy band structures above (Figure 1n,p). Figure 1q shows the calculated results of the bulk modulus and Young’s modulus. The bulk modulus and Young’s modulus of Sb are 38.86 and 33.56 Gpa, respectively, larger than that of Na_3_Sb (15.07, 27.86 Gpa), indicating that the mechanical properties become worse and the materials are more prone to pulverization after the insertion of Na ions, which is mainly due to the fact that the volume of Sb expands about 4.6 times after the sodium ions are embedded based on the DFT calculation. After the introduction of interfacial carbon, the bulk modulus and Young’s modulus of Sb–C and Na_3_Sb–C are 108.8 and 189.5 Gpa larger, and 107.5 and 165.7 Gpa larger than those of Sb and Na_3_Sb, respectively, showing that the deformation resistance can be greatly improved after the interface is introduced during sodium storage, which can effectively prevent material pulverization during the repeated insertion/extraction process of Na ions. These results suggest that the interface can not only improve the charge transfer rate of electrode materials, but can also effectively improve the mechanical properties.

**Figure 2:**
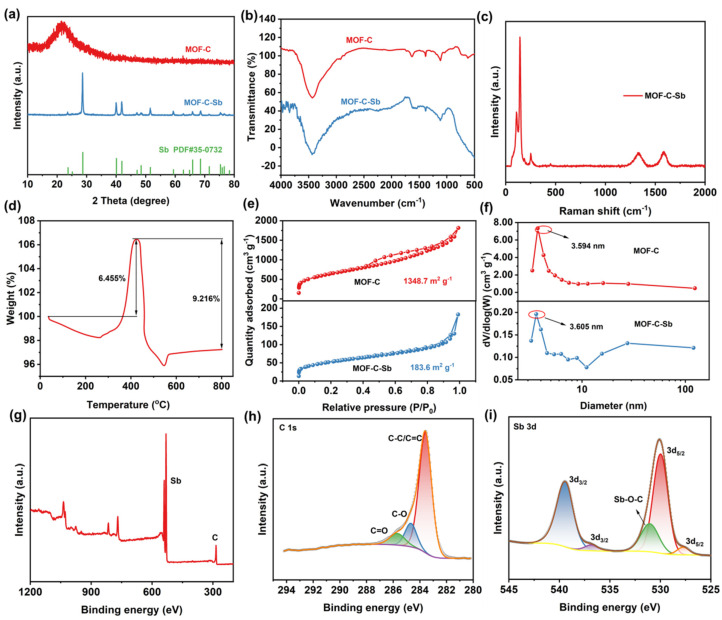
(**a**) The XRD patterns, (**b**) FTIR spectroscopy, (**c**) Raman curves, (**d**) TGA curves, (**e**) N_2_ adsorption−desorption isotherms, (**f**) pore size distributions, and (**g**) XPS survey spectra of the materials. High−resolution XPS spectra of C 1s (**h**) and Sb 3d (**i**) of MOF−C−Sb.

Figure 2a presents the X-ray diffraction (XRD) patterns of MOF-C and MOF-C–Sb. There are two broad peaks at 21.8° and 42.8° for MOF-C, which are characteristic of (002) and (100) crystal planes of amorphous carbon [26,27,28]. The XRD peaks of MOF-C–Sb match the standard card well (Sb, JCPDS card No. 35-0732), indicating successful encapsulation of Sb [7]. Fourier transform infrared (FTIR) spectroscopy was employed to the detection of functional groups in samples (Figure 2b). The absorption peak at ~3400 cm^−1^ is due to the O–H stretching vibration, whereas peaks at ~1633 cm^−1^ and ~1384 cm^−1^ are attributable to the asymmetric and symmetric stretching vibrations of –COO^-^, respectively [29,30]. Significantly, a broader absorption peak appearing below ~600 cm^−1^ is ascribed to the Sb–O bond, suggesting the introduction of Sb. The Raman spectrum of MOF-C–Sb also exhibits characteristic signals of Sb at ∼101 and ∼141 cm^−1^, and two typical peaks at ∼1333 cm^−1^ and ∼1586 cm^−1^ are the D-band and G-band of carbon (Figure 2c). Thermal gravimetric analysis (TGA) of MOF-C–Sb in air has been performed (Figure 2d). The reason for the 2.0% mass loss in MOF-C–Sb at T < 200 °C can be attributed to the loss of water in the as-obtained sample; the weight of composite increases at about 280 °C, which may result from the oxidation of Sb particles (Sb_2_O_3_) in the composite. When the temperature was increased to about 410 °C, the weight was decreased due to the burning of MOF-C; after temperature was increased to 540 °C, the weight increased again by the virtue of the formation of Sb_2_O_4_. After calcining at 800 °C, the Sb species was wholly transferred to Sb_2_O_4_ [31]. Based on this, the weight content of carbon in composite materials is assessed to be 21.4%, which is close to the carbon content of the experimental design. The specific surface area and pore size distribution of MOF-C can be characterized by N_2_ adsorption–desorption. The specific surface area of MOF-C is calculated to 1348.7 m^2^ g^−1^ according to the Brunauer–Emmett–Teller (BET) formula (Figure 2e). Based on the Barrett–Joyner–Halenda (BJH) method, the average pore size of MOF-C is 3.594 nm, implying a large number of micropore/mesopore structures (Figure 2f). The specific surface area and the average pore size of MOF-C–Sb are 183.6 m^2^ g^−1^ and 3.605 nm, respectively, delivering a significant decrease in specific surface area after the loading of Sb, which is conducive to the infiltration of electrolytes. For metal and alloy composite samples, the stoichiometry is particularly important. The deviation from stoichiometry and appearance of the oxygen anions can lead to a change in the charge state of the cations, which in turn will greatly change the electronic parameters. This seriously affects the practical application of the materials obtained [32,33]. The chemical composition and surface oxygen chemical bonding states of MOF-C–Sb are analyzed through X-ray photoelectron spectroscopy (XPS). As depicted in Figure 2g, the XPS survey spectra of Sb and C all exist in MOF-Sb–C. The C 1s high-resolution XPS can be fitted with three subpeaks at roughly 285.8, 284.7, and 283.5 eV, corresponding to C=O, C–O, and C=C, respectively (Figure 2h) [34,35]. As shown in Figure 2i, the Sb 3d high-resolution XPS displays characteristic peaks of Sb 3d_5/2_ (530.0 and 527.7 eV) and Sb 3d_3/2_ (539.5 and 536.7 eV). Moreover, the peak at 531.1 eV is assigned to Sb–O–C, which further illustrates the effective interfacial bonding of Sb in MOF-C–Sb [9,36,37].

**Figure 3:**
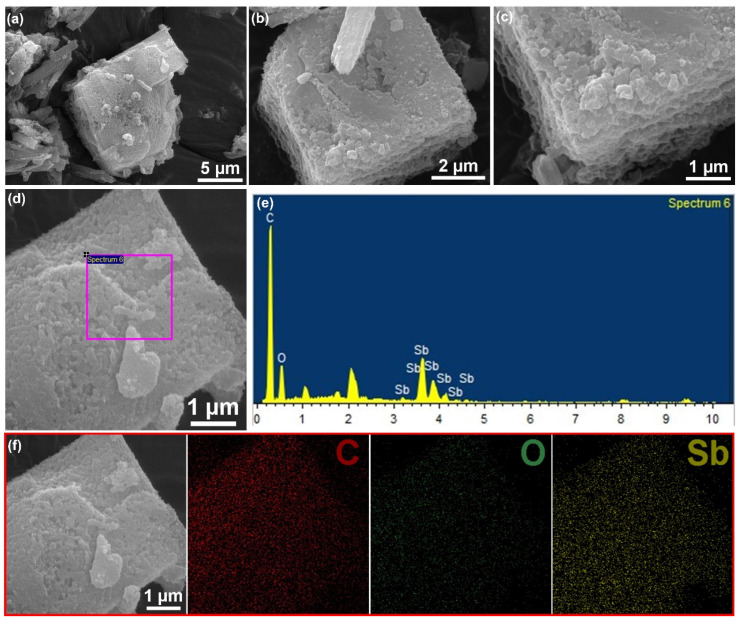
(**a**–**c**) SEM images at different magnifications of MOF-Sb–C. (**d**) The EDX surface scan area and (**e**) the corresponding EDX element results. (**f**) SEM image of MOF-C–Sb and corresponding elemental mapping.

**Figure 4:**
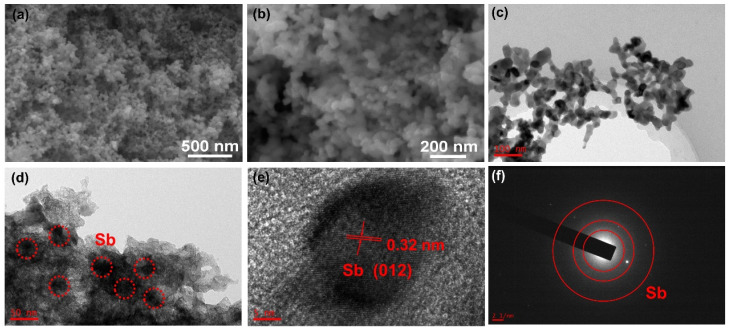
(**a**,**b**) SEM images and (**c**) TEM image of pure Sb. (**d**) TEM image, (**e**) HRTEM image, and (**f**) SAED patterns of MOF-C–Sb.

Figure 3a–c shows scanning electron microscopy (SEM) images of MOF-C–Sb; it can be observed that it exhibits cubic particles with a size of approximately 10 μm. Figure 3d displays a surface scan region of an energy-dispersive X-ray (EDX) image, and the presence of the three elements Sb, C, and O can be clearly observed in Figure 3e. Further, the elemental mappings of MOF-C–Sb confirm the uniform distribution of the three elements Sb, C, and O (Figure 3f). As shown in Figure 4a,b, the SEM images of pure Sb reveal a small granular spherical morphology. Figure 4c shows transmission electron microscopy (TEM) image of pure Sb, exhibiting a small granular spherical morphology, which is consistent with the SEM results. Regarding the TEM image of MOF-C–Sb as shown in Figure 4d, the darker areas within the small red circles are metallic Sb, implying the homogeneous encapsulation of Sb. High-resolution TEM (HRTEM) imagery exhibits a lattice spacing of 0.32 nm for MOF-C–Sb, corresponding to the (221) plane of Sb [7]. In addition, as displayed in Figure 4f, the selected-area electron diffraction (SAED) pattern shows the three main visible diffraction rings of Sb, further demonstrating the successful introduction of Sb.

**Figure 5:**
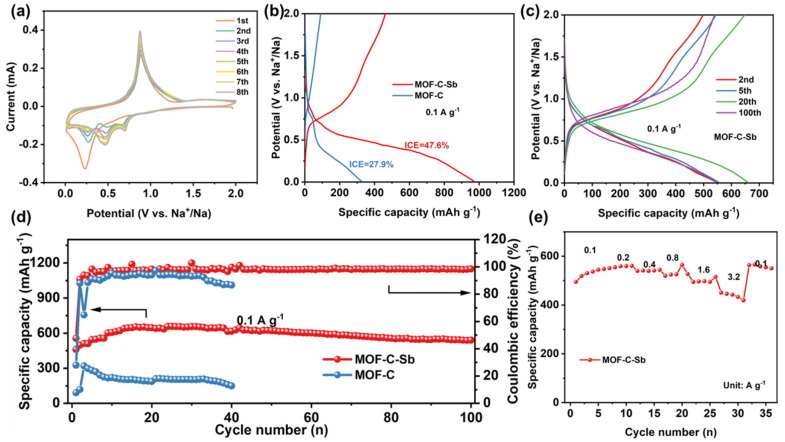
(**a**) CV curves at 0.1 mV s^−1^ of MOF-C–Sb electrode. (**b**) The initial GCD profiles of two different electrodes at 0.1 A g^−1^. (**c**) GCD profiles for MOF-C–Sb electrode during various cycles at 0.1 A g^−1^. (**d**) Cycling performance of the two electrodes at 0.1 A g^−1^. (**e**) Rate performance of the two electrodes from 0.1 to 3.2 A g^−1^.

Figure 5a shows the initial eight cycles’ cyclic voltammetry (CV) curves of the MOF-C–Sb electrode at 0.1 mV s^−1^ between 0.01 and 2.00 V (versus Na^+^/Na) [7]. A sharp peak at 0.23 V is clearly visible in the initial cathodic scan, which could be due to the alloying process of Sb with Na to form Na_x_Sb, the formation of a solid electrolyte interphase (SEI) layer, and some side reactions [38,39,40]. In the next cathode cycles, two new peaks of reduction appear at 0.69 V and 0.46 V, which are attributed to the multi-step conversion from Sb to Na_x_Sb (x ≤ 3) and sodiation to Na_3+x_Sb [8,41]. During the anodic cycles, the strong peak at 0.88 V is caused by the desodiation of Na_x_Sb. The initial galvanostatic charge/discharge (GCD) curves of two electrodes at 0.1 A g^−1^ in the potential range of 0.01–2.0 V are shown in Figure 5b. The capacity loss in the first discharge/charge cycle is mainly related to the irreversible formation of the SEI film. Surprisingly, the initial coulombic efficiency (ICE) of MOF-C–Sb is 47.6%, larger than that of MOF-C (27.9%), implying a remarkable increase in reversible capacity after encapsulation of Sb, which may be attributed to the decreased surface area after loading Sb. MOF-C–Sb has two obvious charge–discharge plateaus corresponding to the alloying process of Sb, which is consistent with the previous CV results (Figure 5c). Moreover, the subsequent charge/discharge curves largely overlap, indicating a high degree of reversibility and stability during the electrochemical cycling processes. The MOF-C–Sb electrode provides a specific capacity of 540.5 mAh g^−1^ after 100 cycles at 0.1 A g^−1^ without obvious capacity degradation, displaying a good cycling stability (Figure 5d). In contrast, the MOF-C electrode displays extremely poor cycling performance, resulting in a capacity of almost 240 after 40 cycles at 0.1 A g^−1^. The results of electrochemical tests confirm that the coating of Sb on MOF-C not only significantly improves the rate performance but also modifies its cycling stability. In addition, the rate capabilities of the MOF-C–Sb electrode at various current densities from 0.1 to 3.2 A g^−1^ in the voltage range of 0.01-2.00 V are illustrated in Figure 5e. The MOF-C–Sb electrode delivers the best rate performance with reversible specific capacities of 547.3, 560.5, 544, 527.2, 515.1, and 419.2 mAh g^−1^ at 0.1, 0.2, 0.4, 0.8, 1.6, and 3.2 A g^−1^, respectively. When the current density returns to 0.1 A g^−1^, the reversible specific capacity can still recover to 550.3 mA g^−1^, demonstrating a superior electrochemical rate capability. All of these results demonstrate the significant role of the heterointerface-engineered structure in enhancing the electrochemical performance for sodium storage.

## 4. Conclusions

In summary, the porous heterointerface MOF-C–Sb with rich Sb–O–C bonds has been successfully fabricated. Density functional theory calculations indicated that interfacial bond engineering can not only largely decrease the band energy and reaction energy barrier of Sb and Na_3_Sb phases, but can also enhance the mechanical properties, exhibiting excellent electrochemical performances for sodium storage. When utilized as an anode for sodium storage, the MOF-C–Sb anode with its rich heterointerface delivers an improved electrochemical performance of 540.5 mAh g^−1^ after 100 cycles at 0.1 A g^−1^, and 515.9 mAh g^−1^ at 1.6 A g^−1^ for sodium storage, efficiently relieving the serious volume expansion issues of metal Sb, demonstrating the structural superiority of the heterointerface-engineered structure and providing a feasible approach to the rational design and construction of Sb-based anode materials for high-performance electrochemical energy storage.
